# Serious gaming and virtual reality in the multimodal training of laparoscopic inguinal hernia repair: a randomized crossover study

**DOI:** 10.1007/s00464-022-09733-6

**Published:** 2022-10-26

**Authors:** Franziska Lang, E. Willuth, C. M. Haney, E. A. Felinska, E. Wennberg, K. F. Kowalewski, M. W. Schmidt, M. Wagner, B. P. Müller-Stich, F. Nickel

**Affiliations:** 1 Department of General, Visceral and Transplantation Surgery, Heidelberg University Hospital, Heidelberg, Germany; 2 Temerty Faculty of Medicine, University of Toronto, Toronto, Canada; 3 Department of Urology and Urological Surgery, University Medical Center Mannheim, Heidelberg University, Heidelberg, Germany; 4 Department of Gynecology and Obstetrics, University Medical Center of the Johannes Gutenberg University, Mainz, Germany

**Keywords:** Laparoscopy, Serious Gaming, Education, Inguinal hernia repair, TAPP, Randomized crossover trial

## Abstract

**Background:**

The aim of this study was to assess the transferability of surgical skills for the laparoscopic hernia module between the serious game Touch Surgery™ (TS) and the virtual reality (VR) trainer Lap Mentor™. Furthermore, this study aimed to collect validity evidence and to discuss “sources of validity evidence” for the findings using the laparoscopic inguinal hernia module on TS.

**Methods:**

In a randomized crossover study, medical students (*n* = 40) in their clinical years performed laparoscopic inguinal hernia modules on TS and the VR trainer. TS group started with “Laparoscopic Inguinal Hernia Module” on TS (phase 1: Preparation, phase 2: Port Placement and Hernia Repair), performed the module first in training, then in test mode until proficiency was reached. VR group started with “Inguinal Hernia Module” on the VR trainer (task 1: Anatomy Identification, task 2: Incision and Dissection) and also performed the module until proficiency. Once proficiency reached in the first modality, the groups performed the other training modality until reaching proficiency. Primary endpoint was the number of attempts needed to achieve proficiency for each group for each task/phase.

**Results:**

Students starting with TS needed significantly less attempts to reach proficiency for task 1 on the VR trainer than students who started with the VR trainer (TS = 2.7 ± 0.6 vs. VR = 3.2 ± 0.7; *p* = 0.028). No significant differences for task 2 were observed between groups (TS = 2.3 ± 1.1 vs. VR = 2.1 ± 0.8; *p* = 0.524). For both phases on TS, no significant skill transfer from the VR trainer to TS was observed. Aspects of validity evidence for the module on TS were collected.

**Conclusion:**

The results show that TS brought additional benefit to improve performances on the VR trainer for task 1 but not for task 2. Skill transfer from the VR trainer to TS could not be shown. VR and TS should thus be used in combination with TS first in multimodal training to ensure optimal training conditions.

For a good surgical outcome, specific knowledge of the anatomy and individual steps of the operation is essential. For this reason, the serious gaming application Touch Surgery™ (TouchSurgery™, Medtronic, London, UK) was developed. Touch Surgery™ (TS) offers a mobile training platform for the simulation of different surgical procedures. Validity evidence for the use of TS in medical education has been collected from multiple specialties. One study demonstrated a correlation between laparoscopic and cognitive skill acquisition between the VR Trainer Lap Mentor™ (LAP Mentor™, 3D Systems, Rock Hill, USA) and the TS application for the laparoscopic cholecystectomy module [1]. Another study evaluated the influence of the application of TS as a training method on chest drain placement and showed a significant improvement in time and motion management in the TS intervention group [2]. The subjects required less assistance and showed more confidence in handling instruments than those in the control group. In an additional study, validity evidence for the use of TS as tool for acquiring cognitive competencies prior to performing surgical procedures in the operating room was collected for the intramedullary femoral nailing module on TS and it was regarded as an effective tool to extend conventional learning methods and showed potential to be included in training curricula [3].

Skills acquired during simulation-based laparoscopic training have been shown to be transferable to the operating setting [4–6]. Simulation training has been proven effective in developing and measuring laparoscopic skills and has qualified as a useful tool in medical education [7–10]. Previous studies have shown that simulation training in surgery could improve medical students’ operative technical skills, patient safety parameters and global performance assessment [11]. It has also been demonstrated that multimodal training, especially combined with practicing skills on VR trainers, helps surgical novices improve their learning curve and operative performance and seems to decrease operating time during laparoscopic training [12–15]. Including VR trainers in surgical training may optimize laparoscopic skill acquisition as it allows for the assessment of individual hand skills, provides automatic feedback that enables competency-based training curricula and permits adapted practice [16,17].

The influence of TS and VR on practical surgical skills acquisition in a multimodal training setting for laparoscopic inguinal hernia repair has yet to be evaluated. There is also still insufficient data available on the validity of the inguinal hernia module on TS.

The aim of the present study was therefore to assess the potential transferability of surgical skills between the laparoscopic hernia module on the serious gaming application TS and the VR trainer Lap Mentor™ III. Additionally, validity evidence using Messick’s framework for the laparoscopic hernia repair module on TS responding to specific questions in the setting of this study was assessed.

## Materials and methods

### Participants

Medical students from Heidelberg University Hospital in their clinical years of study with ten hours of prior basic minimally invasive surgery training experience (*n* = 40) were invited to participate in the study on the potential transferability of laparoscopic skills between TS and VR. Participants with more experience in laparoscopic surgery were excluded. To collect validity evidence of the inguinal hernia module on TS focusing on its use to assess the surgical skill level of trainees, 52 participants were recruited (17 students, 23 surgical residents and 12 surgical senior physicians). Participation was voluntary, and participants were allowed to leave the study at any time. Participants received information about the study and informed consent was obtained. The local ethics committee at Heidelberg University (IRB approval) approved the study protocol before recruitment (S-436/2018).

### Setting and study design

Both studies took place in the training center for minimally invasive surgery at the Department of General, Visceral, and Transplantation Surgery at Heidelberg University Hospital. The study concerning the possible transferability of skills between VR and TS (hereafter referred to as the transferability of skills study) was designed as a prospective single center randomized crossover study. The students were randomized in a 1:1 ratio into two groups, using numbered, sealed, and opaque envelopes. The envelopes were computer-generated by an employee who was not directly involved in in the training, skills testing or data collection. Both groups were compared by the effectiveness of training a laparoscopic inguinal hernia repair. After filling out a questionnaire about baseline characteristics, the participants performed a standardized basic skills training on the VR trainer Lap Mentor™ III and on the application TS as laparoscopic preparation for the inguinal hernia module and to get used to each training platform. The basic skills training on the VR trainer was based on seven basic exercises and was performed twice by each student. After the second run on the VR trainer, phase 2 of the laparoscopic appendectomy was performed on TS, once in training mode and then once in test mode. The basic skills on the VR trainer were analyzed using the established Heidelberger VR score [18].

**Fig. 1:**
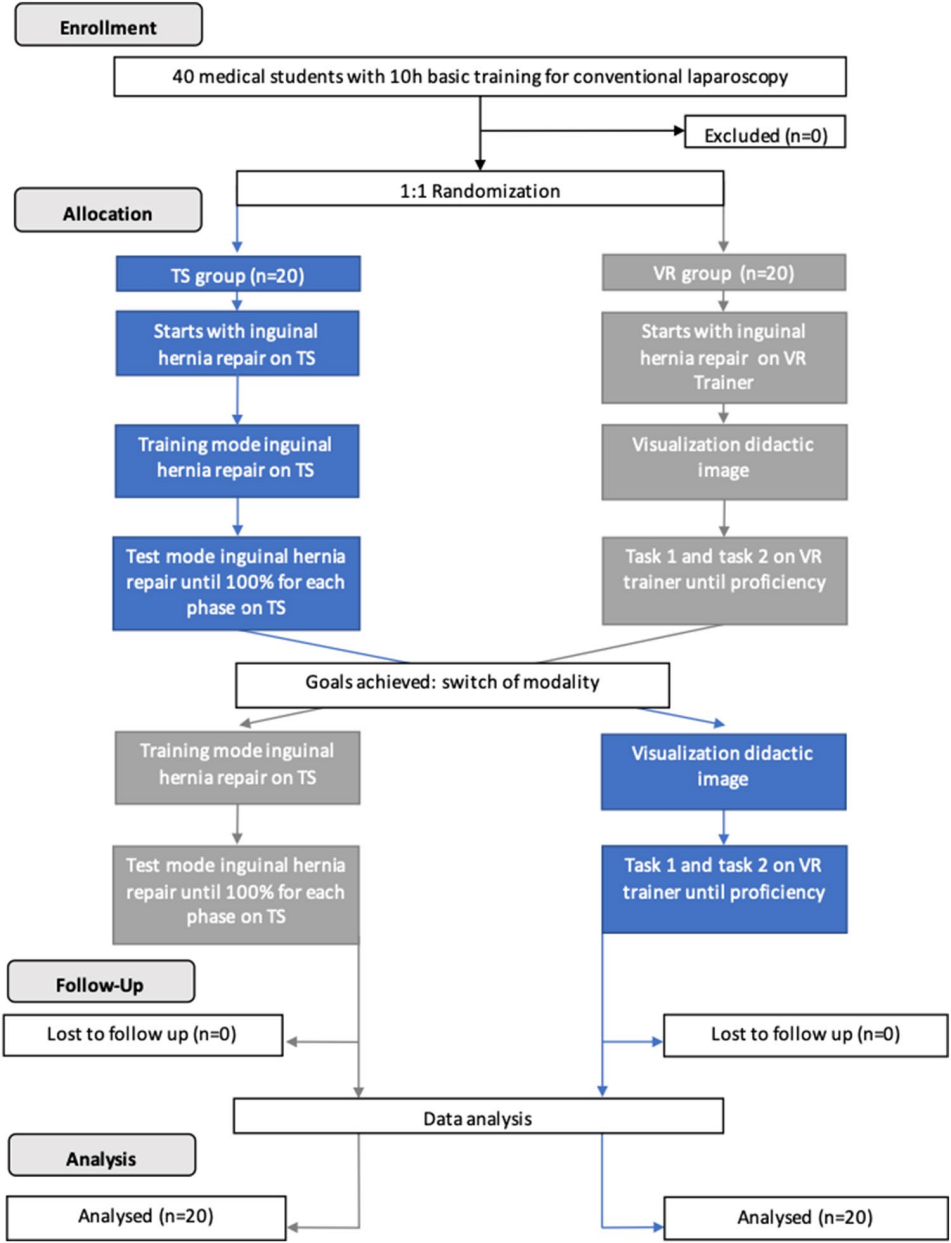
Flowchart for potential transferability of skills study curve comparison between TS and VR group

Each participant performed then the inguinal hernia module on the serious gaming application TS and on the virtual reality (VR) trainer. While performing test mode on TS, students always alternated phase 1 and phase 2. In accordance with the crossover study design, participants in the TS group started with the “Laparoscopic Inguinal Hernia Module” on TS (phase 1: Patient Preparation, phase 2: Hernia Repair) and performed the module first in training, then in test mode until proficiency goals were reached. Proficiency for the laparoscopic inguinal hernia module on TS was defined as a score of 100% in both phases. The VR group started with the “Inguinal Hernia Module” on the VR trainer (task 1: Anatomy Identification, task 2: Incision and Dissection) and also performed the module until proficiency was reached. Proficiency for the inguinal hernia module on the VR trainer was defined for task 1 as correctly identifying every anatomical structure on the first attempt and for task 2 as completing the procedure without complications (injury to vessels or ductus deferens, unstoppable bleeding). Once proficiency goals were reached, the groups switched modalities, and the students performed on the other training modality until the proficiency goals were reached (Fig. 1). For the For the TS validity evidence assessment regarding the use of TS to assess the influence of surgical experience on the performance on the laparoscopic inguinal hernia module on TS (relations to other variables evidence) and if the module on TS was considered useful and representative of the skills needed to learn and perform the actual procedure of laparoscopic inguinal hernia repair (content evidence), all participants performed training mode once for the TS “Laparoscopic Inguinal Hernia Module” and afterward performed test mode, as the procedure was considered advanced, even for surgical residents”.

### Laparoscopic inguinal hernia module

#### Touch surgery™

**Fig. 2:**
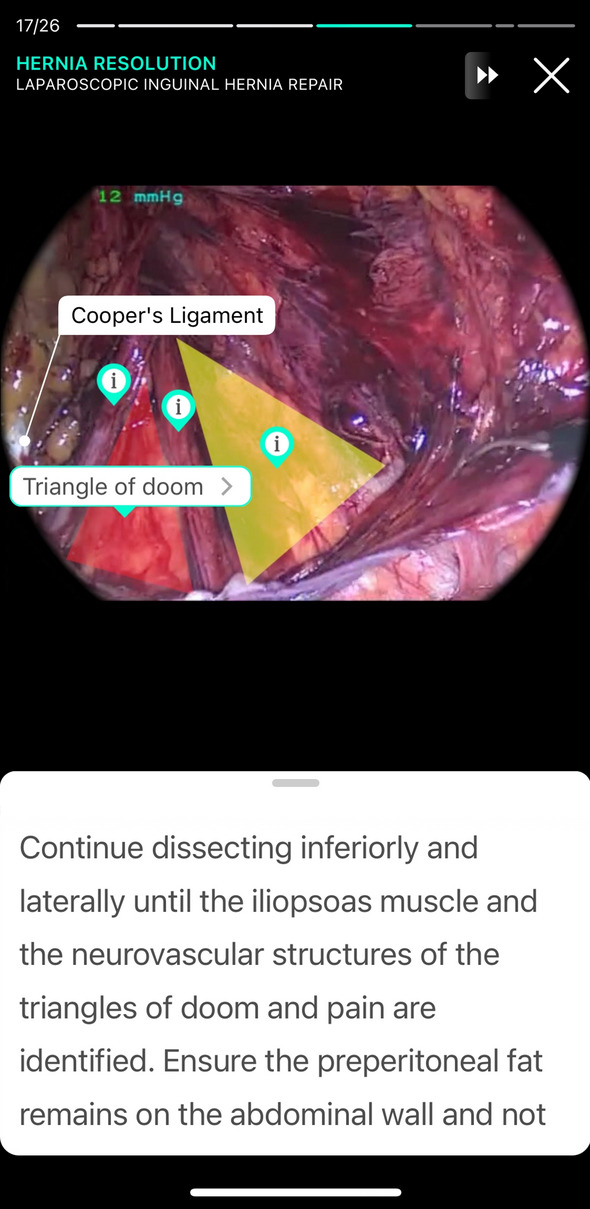
Learning mode for phase 2 on TouchSurgery™ Application for the laparoscopic inguinal hernia repair module

The TS software was installed on the Apple iPad Airs (Apple Inc., Cupertino, California, USA) of the training center for minimally invasive surgery at the Department of General, Visceral, and Transplantation Surgery at Heidelberg University Hospital and were used by the subjects in this setting to complete the module. The module “Laparoscopic Inguinal Hernia Repair” is divided into two phases, phase 1: Preparation (26 multiple choice (MC) questions) and phase 2: Port Placement and Hernia Repair (40 MC questions) (Fig. 2).

#### Lap Mentor III

**Fig. 3:**
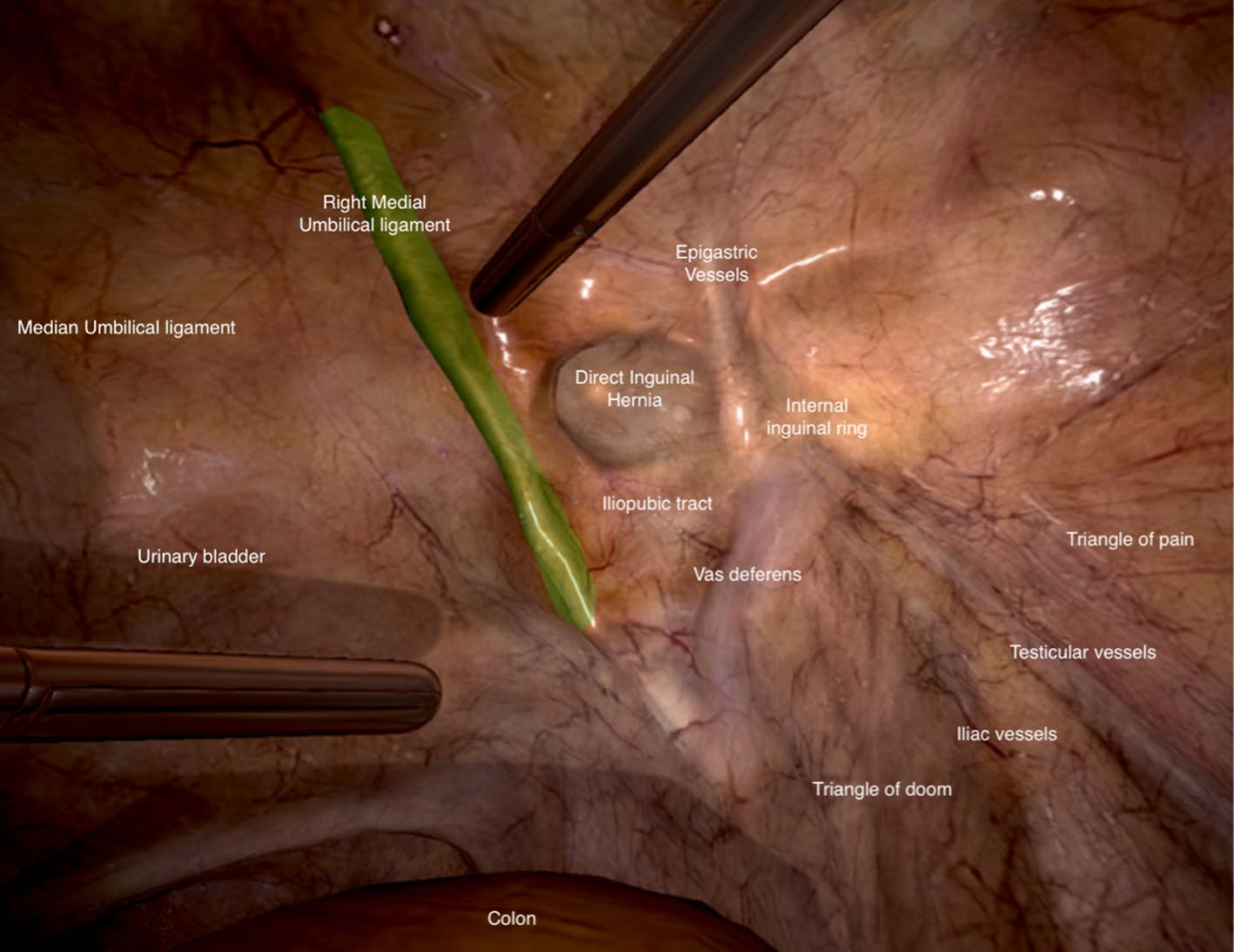
Didactic image for inguinal hernia module for task 1 on virtual reality trainer

All participants completed the “Inguinal Hernia Module” on the same VR trainer, the Lap Mentor III (Lap Mentor™, Simbionix©, Cleveland, USA). The procedure was a Transabdominal Preperitoneal Plasty (TAPP). It is structured into two tasks: task 1: Anatomy Identification and task 2: Incision and Dissection. Participants always completed these two tasks consecutively until proficiency was reached. Proficiency for the inguinal hernia module on the VR trainer was defined for task 1 to correctly identify on the first attempt every anatomical structure and for task 2 to complete the procedure without complications (injury to vessels or ductus deferens, unstoppable bleeding). The number of attempts needed to reach proficiency level was counted, as well as the time needed to perform the procedure (task 2). As the TAPP represents a complex surgical procedure for novices, every student was allowed to look at a didactic image derived from task 1 on the VR trainer (Fig. 3).

### Outcome measures

#### Primary endpoint

In order to compare the potential transferability of skills between both learning modalities (TS and VR) for both groups for the laparoscopic inguinal hernia module, the number of attempts needed to the reach the previously defined proficiency goals for each task/phase on both tools was compared between the TS group and the VR group.

#### Secondary endpoints

As a secondary endpoint, we considered differences between both groups in the operating time needed to perform the inguinal hernia repair during task 2 proficiently. Therefore, operating time for the last attempt was assessed, as it was the same attempt as the one which was counted when students reached proficiency. We also compared TS test mode scores attained in the validation study by participant gender.

### Validity evidence

Validity assessment of the module “Laparoscopic Inguinal Hernia Repair” on the Serious Game Touch Surgery™ was performed based on Messick’s framework. According to Messick, different aspects of validity evidence are combined under the overarching concept of construct validity [19, 20]. The assessment of construct validity is based on five major pillars: content evidence, response process evidence, internal structure evidence, relations to other variables evidence and consequences evidence. However, validity does not refer to the measurement instrument itself, but to the interpretation of the result. According to Messick’s understanding, a tool can never be valid but there can be enough validity evidence to justify the use of a tool and the interpretation of its results for a specific cause [21, 22]. This study focused on some aspects of validity to collect evidence to assess if experience level had an influence on performance on the laparoscopic inguinal hernia module on TS (relations to other variables evidence), and if the module on TS was considered useful and representative of the skills needed to learn and perform the actual procedure of laparoscopic inguinal hernia repair (content evidence).

Content evidence is demonstrated when a tool is considered useful and representative of the skills that need to be learned for the real procedure and when test items and format constitute a relevant and representative sample of the domain of tasks [23–26]. Content validity, as one column of the concept construct validity was obtained using questionnaires. As described in Messick’s Framework, it is suitable to compare experts to novices for assessment of construct validity as their scores vary as expected based on an underlying psychological construct (used when no definitive criterion exists) [26]. A further aspect of construct validity was assessed in this setting by collecting validity evidence regarding relations to other variables. Relations to other variables evidence was assessed by investigating what qualities a test measures, that is, by determining the degree to which these relationships are consistent with the construct underlying the proposed test score interpretations [27, 26]. In this setting, we considered this aspect of construct validity to be proven when the test allowed the differentiation of surgical experience levels between participants [28]. Relations to other variables evidence was collected by comparing test mode performance on TS between students, residents, and senior physicians (association with general level of training) [22].

Therefore, to obtain validity evidence, this study aimed to answer two questions: does experience level influence performance on the laparoscopic inguinal hernia repair module on TS (relations to other variables evidence), and can the laparoscopic inguinal hernia repair module on TS be considered useful and representative of the skills that need to be learned to perform the actual procedure in a simulated environment (content evidence)?”.

### Statistical analysis

The statistical analysis, including the sample size calculation, was done in cooperation with the Institute for Medical Biometry and Computer Science of the University of Heidelberg. Statistical analyses and descriptive statistics were performed with SPSS version 25.0 (IBM SPSS Inc., Chicago, Illinois, USA). Data were given as absolute frequency and as mean ± standard deviation. The evaluation was carried out under consideration of the crossover design [29].

Differences between the TS and VR groups were assessed for parametric data using the *t*-Test for independent samples and for non-parametric data using the Mann–Whitney-*U* test for independent samples. Multivariate regression was used to evaluate whether demographic and personal characteristics had an influence on students’ surgical performance while performing the laparoscopic inguinal hernia module on TS and VR. To assess differences between students, residents, and senior physicians for the validation study, the Kruskal–Wallis test was used while assessing non-parametric data (content evidence) and parametric data (relations to other variables evidence). Linear regression was used to assess whether gender had an influence on TS test mode scores obtained during the construct validity assessment. A *p*-value of *p* < 0.05 was considered statistically significant.

## Results

### Demographics

In total, 40 medical students participated in the randomized crossover study (20 per group). All 40 students completed the entire study protocol between June and October 2019. No significant differences were observed between groups for baseline characteristics (Table 1).

**Table 1:** Participants’ baseline characteristics stratified by group (*n* (%))

	TS group (*n* = 20)	VR group (*n* = 20)
Sex (male)	12 (60%)	10 (50%)
Age (years)	22.0 ± 1.0	22.0 ± 1.0
Medical school module of surgery completed	8 (40%)	10 (50%)
Dominant hand (right)	19 (95%)	14 (70%)
Video game activity (yes)	15 (75%)	11 (55%)
Sports activity (yes)	19 (95%)	18 (90%)
Playing a musical instrument (yes)	16 (80%)	15 (75%)
Use of E-learning platforms (yes)	15 (75%)	12 (60%)
Use of online teaching videos for surgery (yes)	9 (45%)	6 (30%)

#### Multivariate regression analysis regarding surgical performance in dependance of demographics

Students’ demographic and personal characteristics, including gender (*p* = 0.058), age (*p* = 0.457), sports activities (*p* = 0.584), playing a musical instrument (*p* = 0.267) and even playing video games (*p* = 0.490), showed no influence on their surgical performance on the TS and VR laparoscopic inguinal hernia modules. Visualizing online teaching videos for surgery (*p* = 0.489) and using E-learning platforms (*p* = 0.387) also showed no effect on students’ performance in the TS and VR modules.

### Primary outcome

#### Transferability of skills: number of attempts needed to reach proficiency for TS and VR trainer

**Fig. 4:**
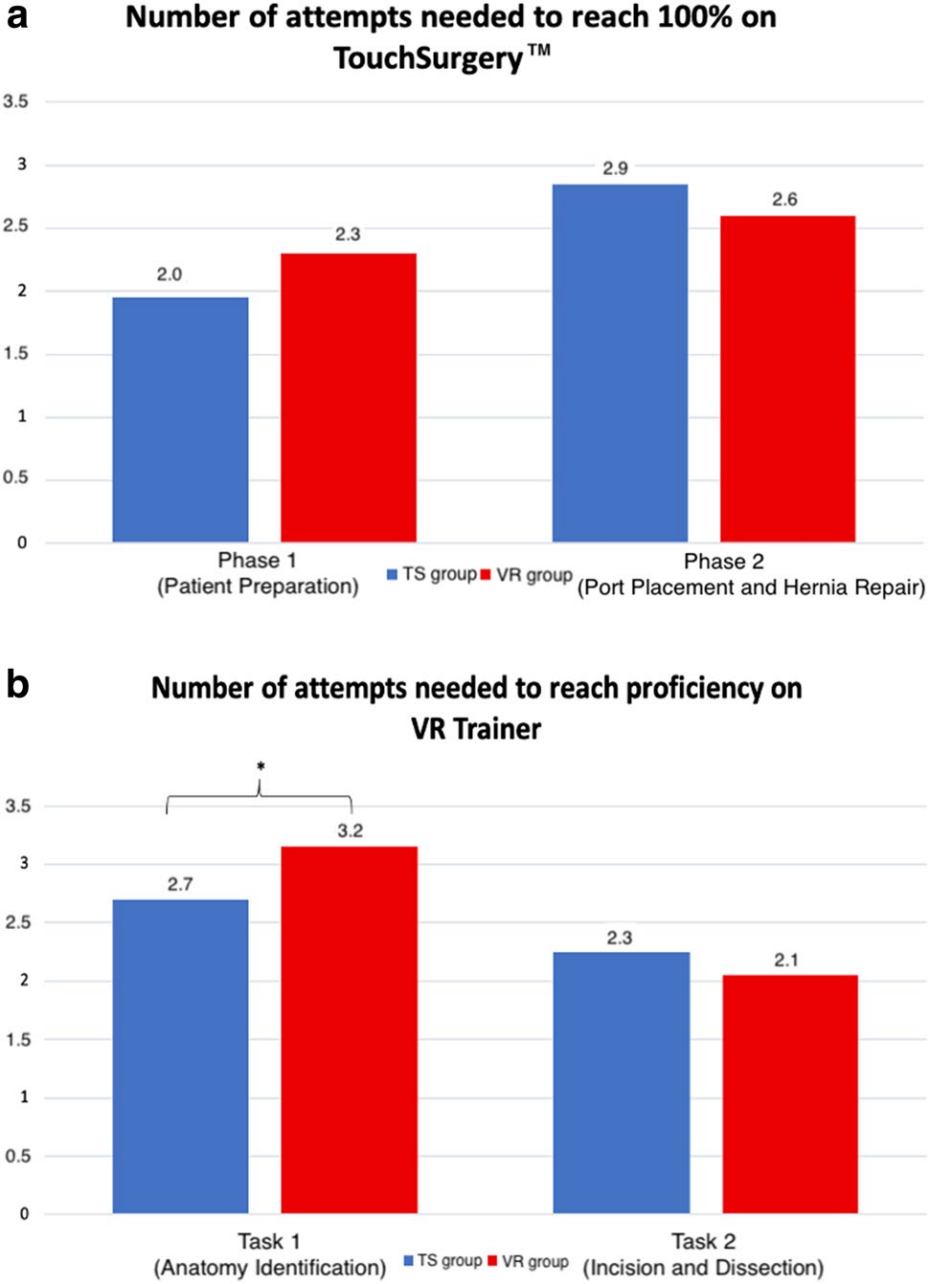
**a** Primary endpoint (number of attempts needed to reach 100%) on Touch Surgery.™ (TS) between groups. **b** Primary endpoint (number of attempts needed to get proficient on virtual reality (VR) Trainer). Independent *t*-Test and Mann–Whitney-*U*, *significant for a *p* < 0.05

Students who started with TS needed significantly less attempts to reach the predefined goal for task 1 on the VR trainer than students who started with the VR trainer (TS group = 2.7 ± 0.6 vs. VR group = 3.2 ± 0.7; *p* = 0.028). No significant differences for task 2 were observed between the two groups (TS group = 2.3 ± 1.1 vs. VR group = 2.1 ± 0.8; *p* = 0.524). For both phases on TS, no significant skill transfer from the VR trainer to TS was observed (phase 1: TS group = 2.0 ± 0.5 vs. VR group = 2.3 ± 0.7; *p* = 0.157, phase 2: TS group = 2.9 ± 0.8 vs. VR group = 2.6 ± 0.8; *p* = 0.253). The results are summarized in Fig. 4a and b. The median number of attempts needed to reach proficiency for task 1 on the VR trainer showed similar results for both groups (TS group 3 [2;3] vs. VR group = 3 [3;3]).

### Secondary endpoints

There were no differences in the time needed to complete task 2 proficiently between the TS and VR groups (TS group (min) = 16.8 ± 5.2 vs. VR group 17.6 ± 8.4; *p* = 0.715). Linear regression showed that gender had no significant influence on the test mode scores reached in % during the validation study in both phases on TS (Table 2).

**Table 2:** Linear regression analysis over gender distribution and scores on TS during validation study

Mean ± SD	Male (score reached on TS in %)	Female (score reached on TS in %)	Regression coefficient	Correlation coefficient *R*	*R*^2^	*p*-value
Patient preparation	90.3 ± 6.4	90.3 ± 9.4	− 0.005	0.000	0.000	0.998
Port placement and hernia repair	82.0 ± 9.2	84.2 ± 9.2	2.223	0.120	0.015	0.395

### Validity evidence

This study collected validity evidence to justify the use of the laparoscopic inguinal hernia repair module on TS in training curricula regarding its usefulness and representativeness of the skills that need to be learned to perform the actual procedure (content evidence) and in assessing potential differences in performance depending on experience level (relations to other variables evidence). The module was rated positively by all three groups and ratings showed that all participant categories found the module useful overall, but some points were rated slightly differently between the three groups. Senior physicians showed significantly more criticism regarding the personal use on mobile devices, found the serious game less fun and were less enthusiastic than students and residents regarding its use. Residents and students answered most of the questions as likely. Both residents and students rated the use of serious games better than senior physicians. Students and residents also considered the possibility of mobile use as more important and useful than senior physicians. Regarding relations to other variables evidence, the collected evidence on the performance on TS for both phases showed no significant variations in scores between students, residents, and senior physicians for the laparoscopic inguinal hernia module on TS. The results are shown in Table 3”.

**Table 3:** Relation to other variables validity (score reached on the first attempt in %), and content evidence

Mean ± SD	Students (*n* = 17)	Residents (*n* = 23)	Senior physician (*n* = 12)	*p*-value
Relations to other variables evidence (as part of construct validity)				
Patient preparation	87.4 ± 10.4	91.7 ± 5.8	91.7 ± 5.7	0.493
Port placement and hernia repair	82.9 ± 9.1	81.6 ± 9.3	85.3 ± 9.1	0.654
Overall	85.1 ± 9.9	86.6 ± 9.2	88.5 ± 8.1	0.483
Content evidence (as part of construct validity)				
Touch Surgery is a useful training modality	4.4 ± 0.9	4.3 ± 0.6	4.1 ± 0.9	0.452
Touch Surgery is helpful to train this operation	4.1 ± 1.0	4.1 ± 0.7	3.8 ± 0.9	0.557
Touch Surgery should be used more often	4.1 ± 1.0	4.3 ± 0.6	3.6 ± 0.9	**0.038**
Through the training with Touch Surgery, I feel prepared for the operation	3.1 ± 1.1	3.5 ± 1.1	2.9 ± 1.0	0.220
This training I more effective than with specialized books	4.1 ± 1.1	4.1 ± 0.9	4.0 ± 0.6	0.676
Through the training with Touch Surgery, I DONT feel adequately prepared	2.5 ± 1.2	2.4 ± 1.0	2.6 ± 1.0	0.928
Residents should train on Touch Surgery before performing for their first in the operating room	4.2 ± 0.8	3.8 ± 1.0	3.4 ± 1.2	0.103
I will use Touch Surgery also on my phone/tablet	3.5 ± 1.4	4.0 ± 1.1	2.1 ± 1.2	**0.001**
The possibility of using Touch Surgery on a mobile device is important	4.7 ± 0.6	4.5 ± 0.9	3.7 ± 1.1	**0.007**
Touch Surgery is a use full supplement to the existing learn methods like books, E-learning, Box Trainer and computer simulations with virtual reality	4.8 ± 0.8	4.6 ± 0.6	4.1 ± 0.7	**0.005**
Touch Surgery is appropriate to test knowledge	3.8 ± 1.0	3.8 ± 1.1	3.6 ± 0.7	0.534
Touch Surgery is appropriate to gain knowledge about the operation specific aspects	4.0 ± 0.9	3.9 ± 0.8	3.4 ± 1.0	0.176
Touch Surgery is an effective tool to learn cognitive skills	3.6 ± 1.2	4.0 ± 1.1	3.8 ± 0.8	0.597
Touch Surgery is an effective tool to learn laparoscopic skills	2.6 ± 1.5	2.8 ± 1.2	2.1 ± 0.7	0.278
E-learning				
Learning with Touch Surgery is fun	4.2 ± 0.8	4.1 ± 0.8	3.3 ± 0.9	**0.014**
I think learning with Touch Surgery is good	4.4 ± 0.7	4.3 ± 0.7	3.4 ± 0.9	**0.006**

Kruskal–Wallis test. This questionnaire used a 5-point Likert scale (1: I do not agree, 5 I totally agree)

Statistically significant *p* < 0.05 values are given in bold

### Advantages, disadvantages and helpful aspects of the application TS

Advantages commonly listed by students were the possibility of mobile use/free availability of the application and its interactivity. Residents considered the visual representation/animation and the step-by-step learning of the procedure as most advantageous. Senior physicians’ opinions were substantially more diverse, and they noted realism, step-by-step learning of the procedure, visual representation/animation and interactivity as equal major advantages. The major disadvantage named by students was that TS does not provide practical training. Common disadvantages named equally by residents were insufficient representation/information and there being only one standard (USA). Senior physicians equally named insufficient representation/information and the lack of practical training as disadvantages. In terms of skills that could be acquired using TS, all three groups highlighted provision of knowledge about the procedural steps as the most helpful aspect of the application.

## Discussion

The present study assessed the potential transferability of surgical skills between the laparoscopic hernia modules on TS and on the VR trainer LapMentor™ III. Furthermore, some validity evidence for the laparoscopic hernia repair module on TS was collected. This study showed that TS provided additional benefit for students to improve their skills regarding anatomical knowledge about the procedure of laparoscopic inguinal hernia repair on the VR trainer for task 1. However, results showed no skill transfer from TS to the VR trainer for task 2, nor from the VR trainer to TS for either phase. There were no differences between groups in operating time needed to perform task 2 on the VR trainer proficiently. Content evidence could be collected for the module “Laparoscopic Inguinal Hernia Repair” on the serious game TS as the module was rated positively regarding usefulness by the participants. As a further aspect of construct validity, some evidence regarding relations to other variables was collected. In fact, as there were no score differences between students, residents, and senior physicians, the interpretation of this result shows that experience level had no influence on performance on the laparoscopic inguinal hernia repair module on TS.

Students from the TS group started with the module on TS and performed afterward on the VR trainer, and students from the VR group performed on the VR trainer first and then on TS. Students from the TS group needed significantly less attempts to reach the predefined goal for task 1 (Anatomy Identification) on the VR trainer than students from VR group. One cannot strictly speak of skill transfer between TS and VR as the two modalities test different competencies. TS puts focus on theoretical knowledge of procedures, and the Lap Mentor tests practical skills. Nevertheless, prior performance of the module on TS led to an additional benefit as it helped students increase their performances on the VR simulator regarding anatomical skills. For task 2 on the VR trainer, no significant differences were observed between the groups and so, no skill transfer from TS to the VR trainer took place for task 2. There were also no differences in the time needed to complete task 2 proficiently between the TS and VR groups. Indeed, TS provides purely theoretical training which could help students perform better during task 1, as this task requires anatomical knowledge. Task 2 on the other hand requires practical skills, as students had to perform the incision and peritoneal dissection steps of the TAPP. TS therefore provided theoretical knowledge about anatomy which helped to improve student’s skills on the VR trainer. So, TS can be considered a useful tool to supplement and improve surgical laparoscopic training. This has also been shown in previous educational research in this field [30–32]. VR used in a multimodal setting may also supplement training curricula in surgical education, as VR simulation has already been demonstrated to be effective and useful [33, 34]. VR and TS should thus be used in a multimodal training setting to ensure optimal training conditions, with TS preceding VR.

For both phases on TS, students in both groups showed similar numbers of attempts needed to reach a score of 100%. Therefore, no significant skill transfer from the VR trainer to TS was observed. This is to be expected as the laparoscopic hernia module on the VR trainer teaches purely practical knowledge about the procedure, and the MC questions used in test mode on TS are based on theoretical aspects of the procedure. Furthermore, phase 1 on TS tests knowledge about patient preparation, which is not taught in the VR trainer module. Additionally, the mesh placement component of task 2 on the VR trainer was still under development while conducting this study, so students were trained only for the peritoneal preparation. Phase 2 on TS tested knowledge about the whole procedure, including mesh placement. Previous research has shown similar results; one study showed no additional benefit from the VR trainer in a multimodal training setting [35]. Students’ demographic and personal characteristics regarding gender, age, performing activities like sports, playing an instrument, playing video games, visualizing videos for surgery, or using E-Learning platforms unexpectedly showed no significant influence on the students’ surgical performance.

This study collected some evidence for construct validity based on the assessment of content evidence and relations to other variables evidence. Content evidence was gathered for the module “Laparoscopic Inguinal Hernia Repair” on the serious game TS as it was positively rated by all three groups. The possibility of mobile use was considered by students to be an advantage of the program and it may allow take-home training as users can freely access the application anytime. Take-home training has been shown to be an effective training method even if unsupervised and can enable higher training frequency, as session duration can be chosen freely. Even self-rating by the user has been considered as reliable [36]. The application provides flexible training with good cost effectiveness [37]. The module on TS can be considered suitable to respond to its purpose, as the question “can the laparoscopic inguinal hernia repair module on TS be considered useful and representative of the skills that need to be learned to perform the actual procedure?” was answered positively in the questionnaires completed by the surgeons. Therefore, content evidence was collected which supports the use of the module “Laparoscopic Inguinal Hernia Repair” on TS in surgical training curricula [38]. Regarding relations to other variables, the collected evidence reflects that the experience level of the participants had no veritable influence on the performance on TS for the laparoscopic inguinal hernia module as no significant variations in scores were observed between students, residents, and senior physicians for either phase. The question “does experience level influence performance on the laparoscopic inguinal hernia repair module on TS?” was answered negatively, nevertheless this result shows some evidence for the module on TS. Therefore, the learning mode helped novices to acquire sufficient competencies and theoretical knowledge to perform at a level comparable to surgeons and to overcome the difference in experience level for this procedure on TS. This contradicted our expectations, as previous studies showed differences in the participants’ performance depending on their experience level for other TS modules [39–41]. This could be explained by the fact that all participants performed the training mode once before performing test mode, due to the high complexity of the procedure for students and even for residents, and because some of the surgeons (residents and senior physicians) did not agree with some procedural steps as the operating technique taught on TS was considered slightly different from the technique commonly used at Heidelberg University Hospital, especially regarding mesh placement or suturing technique. Nevertheless, the interpretation of the results shows evidence toward construct validity that justifies the use of TS in laparoscopic training regarding its usefulness and representativeness of the skills that need to be learned to perform the actual procedure (content evidence) and the fact that TS taught the novices sufficient theoretical competence to allow performances on test mode comparable to experts’ performances (relations to other variables evidence). Further assessments are necessary, as this setting only focused on some aspects of the concept validity and can only be applied to the laparoscopic inguinal hernia module on TS regarding our specific questioning.

An additional factor which could explain why surgeons and especially senior physicians did not score significantly higher than students was that most of them participated after long working hours in the operating room and showed more signs of tiredness and drops of concentration and motivation than students. Furthermore, students are more accustomed to the MC format of the TS test mode as they still use it on a regular basis and read through the training mode more slowly and carefully as the surgeons before taking test mode as they were usually less pressed for time. Finally, some senior physicians criticized the fact that the module is only available in English and showed some linguistic barriers.

## Limitations

As the TS module was only available in English, this could be seen as a limitation to the study. Some of the senior physicians noted differences in terminology between German and English and showed struggles in responding to the MC questions as a result. Also, the version of TAPP taught by TS differed slightly in operating technique compared to the Heidelberg standard. This was highlighted by most of the surgeons (residents and senior physicians) as it led them to answer some test questions falsely because they disagreed with the operating method. Nevertheless, every participant performed the learning mode on TS for both phases before performing on test mode, so they could familiarize themselves with the terminology. Additionally, comparing the transferability of laparoscopic skills between the inguinal hernia modules on TS and the VR trainer showed limitations in this study as the module on the VR trainer taught and tested skills on anatomy, incision, and peritoneal dissection, but not mesh placement, as this component was still under development at the time this study was conducted. Mesh placement forms part of the TAPP procedure and was taught and tested on TS. Furthermore, rather than talking about skill transfer from TS to the VR trainer, the results of this study should be interpreted as showing the additional benefit of using Serious Games in combination with VR to increase the performance on the VR trainer regarding anatomical knowledge.

The transferability to clinical performance was not assessed, as this study could not assess the actual performance in real patients. Nevertheless, our study enabled the assessment of the value of Serious Gaming and VR in current training curricula for MIS”. Transferability of simulation training to the clinical praxis has been demonstrated previously; studies have shown the benefit of simulation-based training outside the OR for surgical performance, as the skills acquired during training helped to improve intraoperative performance and enhance patient outcomes. In the training of laparoscopic TEP inguinal hernia repair, simulation-based training has been shown to improve trainee performance, decrease operative time, and reduce intra- and postoperative complications as well as overnight stays [42]. Training outside the OR allows trainees to acquire surgical skills that help them to train key steps of surgical procedures and prepares them for the training phase in the operating room [43].

## Conclusion

TS led to an additional benefit in the form of improved performance on the VR trainer for task 1 (Anatomy Identification) but not for task 2 (Incision and Dissection). This can be explained by the fact that TS provides only theoretical training and task 1 on the VR trainer tests anatomical knowledge, while task 2 tests practical skills. Skill transfer from the VR trainer to TS could not be shown. This can be explained by that fact that the VR trainer module taught mostly practical skills. Also, the VR trainer taught only the peritoneal preparation (mesh placement was still under development during the study) and did not teach patient preparation. The study shows some evidence for construct validity regarding content and relations to other variables evidence for the “Laparoscopic Inguinal Hernia Repair” module on the serious game TS. VR and TS should thus be used in combination in multimodal training, ideally with TS being used first to ensure optimal training.

## Publication related to this study

**Fig. 5:**
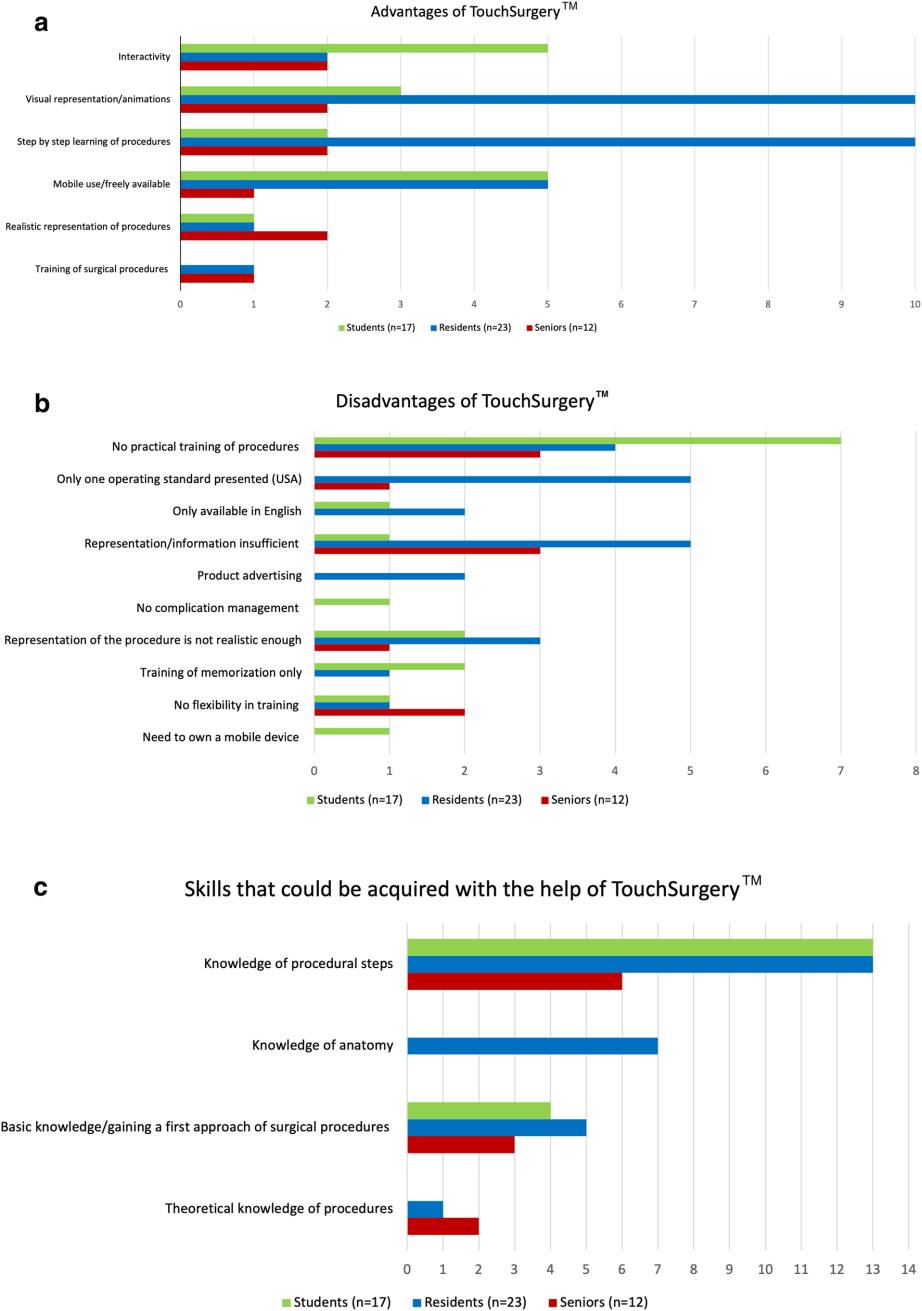
**a** Advantages of Touch Surgery™. **b** Disadvantages of Touch Surgery™. **c** Skills that could be acquired with the help of Touch Surgery™. Absolute number of responses

The Doctoral thesis at Heidelberg University Medical School “Intermodale Übertragbarkeit laparoskopischer Fertigkeiten im Training der Minimalinvasiven Chirurgie” by Estelle Willuth includes content of the present study. This refers to results of the present study which assessed the potential transferability of skills between TS and VR for the laparoscopic inguinal hernia repair module and aimed to validate the laparoscopic hernia module on TS. It concerns Figs. 2, 3, 4a, 4b, 5a-5c as well as Tables 1, 2, and 3 presented in the present study.

## Abbreviations

TS: Touch Surgery™
VR: Virtual reality
MIS: Minimally invasive surgery
MC: Multiple choice
OR: Operating room
